# Genetic diagnosis of the Ehlers-Danlos syndromes

**DOI:** 10.1515/medgen-2024-2061

**Published:** 2024-12-03

**Authors:** Johannes Zschocke, Serwet Demirdas, Fleur S. van Dijk

**Affiliations:** Medical University Innsbruck Institute of Human Genetics Peter-Mayr-Str. 1 6020 Innsbruck Austria; Erasmus Medical Centre Department of Clinical Genetics Dr. Molewaterplein 40 3015 Rotterdam Netherlands; London North West University Health Care NHS Trust National EDS service Watford Road HA1 3UJ Harrow United Kingdom

**Keywords:** Ehlers-Danlos syndrome, EDS, gene panel, hypermobility, skin hyperextensibility, skin fragility, organ rupture, genetic testing

## Abstract

The Ehlers-Danlos syndromes (EDS) represent a group of genetically diverse disorders characterized by the variable combination of joint hypermobility, hyperextensibility of the skin, and connective tissue fragility affecting the skin and other organs. Based on clinical features, 13 different types of EDS have been delineated, 12 of which represent monogenic conditions caused by pathogenic variants in 21 confirmed genes. Pathogenesis is related to disturbances of collagen formation and/or stability. No monogenic cause has been identified for hypermobile EDS (hEDS), a more common EDS type, which is unlikely to represent a single gene disorder in the majority of affected individuals and at present cannot be diagnosed by genetic investigations. Here we summarize the clinical features and the molecular bases of the monogenic EDS types, highlight diagnostic challenges, and provide guidance for the molecular work-up of affected individuals. In general, genetic tests are indicated if clinical features suggest a monogenic EDS type but are usually unrewarding for other cases of hypermobility.

The Ehlers-Danlos syndromes (EDS) represent a group of genetically diverse disorders characterized by the variable combination of joint hypermobility (with complications such as recurrent dislocations and chronic joint pain), hyperextensibility of the skin, and connective tissue fragility affecting the skin and other organs. Most types of EDS result from deficiency or alteration of collagens or associated extracellular matrix (ECM) proteins [27]. The 2017 International Classification of the Ehlers Danlos syndromes defined 13 different EDS types linked to 20 different genes [29]. One additional genetically defined entity that may be grouped in the classical-like EDS (clEDS) category has been reported since [5], with another entity not yet fully confirmed [21].

The EDS group of disorders can be divided into “monogenic EDS types” caused by pathogenic variants in specific genes, and hypermobile EDS (hEDS) with yet undefined molecular cause. Most monogenic EDS types – including those with immediate management consequences – show specific clinical features that enable efficient and effective genetic testing. In 2017, strict criteria for a diagnosis of hypermobile EDS were formulated also to facilitate gene discovery in this group of patients. In contrast, the two major diagnostic criteria for hEDS pre-2017 – (1) generalised joint hypermobility (Beighton score ≥ 5) and (2) hyperextensible and/or smooth, velvety skin – were less strict, leading to the diagnosis of “EDS” in a large number of individuals without currently identifiable genetic cause. There is general agreement that individuals diagnosed with hEDS pre-2017 can retain this diagnosis, but in the present manuscript the term hEDS refers to adults diagnosed with hEDS according to the 2017 criteria. It has been recognized that the 2017 hEDS criteria are not suitable for children [42].

Hypermobile EDS overlaps with hypermobility spectrum disorder (HSD), and it may be argued that hEDS/HSD represents a phenotype spectrum with similar – highly variable – manifestations and therapeutic challenges for affected individuals. Apart from general joint hypermobility and subsequent complications, individuals with hEDS can have systemic manifestations of a generalized connective tissue disorder such as abdominal hernias and/or uterine or bowel prolapse, which are also not infrequent in the general population; there may be mild aortic dilation but rarely arterial ruptures. The skin can be soft, velvety and/or mildly hyperextensible with marked striae and mild atrophic scarring, but there is generally no significant skin fragility. Additional sometimes debilitating features are chronic musculoskeletal pain (not sufficiently explained by degenerative joint changes), chronic fatigue, sleeping disturbances, dysautonomia, bowel disease without known pathogenic mechanism, and depression [41]. hEDS and HSD taken together have an estimated incidence of up to 1:500 [15]. The number of individuals with hEDS/HSD substantially exceeds the combined number of individuals with all the known monogenic EDS types. Making the correct clinical diagnosis in individuals with “syndromic hypermobility” – i. e. hypermobility combined with variable other disease manifestations – is a major challenge as the diagnosis of hEDS is primarily clinical and cannot be confirmed with a genetic test or another laboratory investigation.

A broad massively parallel sequencing “EDS panel” is sometimes recommended to exclude a genetic diagnosis in individuals with hypermobility even if there is no clear clinical suspicion of a monogenic disease. Although a pathogenic variant in one of the “EDS genes” is sometimes identified in these cases, this non-specific approach may lead to an excess of expensive genetic investigations with unproven utility. It also has the risk of identifying potentially misleading variants of unknown significance (VUS) that may cause undue uncertainty and sometimes incorrect diagnoses, trigger unnecessary follow-up tests, and may lead to inadequate treatments.

Depending on health system resources, it is preferable to avoid genetic tests in suspected hEDS, or provide these to selected individuals that fulfil the strict 2017 hEDS criteria when the indication for testing is made by specialist clinical geneticists or rheumatologists with experience in diagnosing EDS. Vice versa, in the absence of pathognomonic monogenic EDS manifestations in individuals with hEDS/HSD, the latter diagnosis may be made based on the clinical assessment without the need to “exclude” a monogenic EDS type. In this article, we summarize the main features of the different monogenic EDS types and highlight pitfalls in the genetic diagnosis.

## Major monogenic EDS types

### Classical EDS (cEDS)

cEDS represents the paradigmatic form of the Ehlers-Danlos syndromes, with the defining features of generalized joint hypermobility, marked hyperextensibility of generally soft, doughy skin, as well as skin fragility with poor wound healing and atrophic scarring [6]. Many affected individuals suffer from chronic musculoskeletal and joint pain. Additional features include easy bruising with lasting hemosiderin deposits and other skin signs such as molluscoid pseudotumors, subcutaneous spheroids and piezogenic papules. There may be a history of hernias and mild aortic dilatation, but aortic rupture and other serious cardiovascular events are uncommon.

With an estimated incidence of approximately 1:20 000 [27], cEDS is the most common monogenic form of EDS. It is usually caused by deficiency or structural abnormality of type V collagen, a heterotrimeric fibrillar collagen that forms the scaffold on which type I collagen is deposited for fibril formation (homozygous loss of *COL5A1* expression is embryonic lethal with no normal collagen fibrils formed) and plays an important role in the assembly and regulation of fibrillogenesis. Inheritance is autosomal dominant; more than 90 % of individuals with cEDS carry heterozygous pathogenic variants in one of the two type V collagen genes, *COL5A1* or *COL5A2*. The majority of individuals with cEDS have pathogenic variants in *COL5A1,* both loss-of-function (LoF, null) and missense (structural) variants without evidence of major phenotypic differences. *COL5A2* variants are generally missense/structural and may be associated with a more severe clinical manifestation [11, 38]. Rarely a cEDS phenotype may be due to specific variants in type I collagen genes, in particular arginine-to-cysteine variants which may be associated with an increased probability of vascular events. The main differential diagnosis is classical-like EDS (clEDS); some individuals with hEDS show skin manifestations that may resemble cEDS, but careful clinical evaluation usually allows reliable distinction between the two conditions.

Genetic diagnosis of cEDS involves complete sequence analysis plus deletion/duplication testing of *COL5A1* or *COL5A2* as well as *COL1A1* and *COL1A2,* usually by massively parallel sequencing. It must be noted that most pathogenic variants in the type I collagen genes cause osteogenesis imperfecta, and the EDS phenotypes associated with variants in these genes may have some specific clinical features. In the case of a heterozygous VUS in one of the *COL5A1/2* genes, segregation analysis in the family, skin electron microscopy, or functional/transcript studies, may provide additional diagnostic clues. Non-transcription of one copy of a collagen V gene may also be shown by loss of heterozygosity in transcript analyses in individuals with a heterozygous polymorphism on DNA level (also called null-allele test) [28, 39, 44].

### Vascular EDS (vEDS)

Vascular or organ rupture is the hallmark of vEDS, the most life-threatening form of EDS [8]. It has an estimated incidence of 1:50 000–1:200 000 [27]. vEDS should be considered in all individuals with arterial rupture, dissection or aneurysm before age 40 years, otherwise unexplained bowel or uterine rupture, or carotid-cavernous sinus fistula without previous trauma. Hypermobility is often discrete and limited to the small joints. The skin is often thin and translucent with marked venous visibility and general acrogeric appearance; there may be bruising at unusual body sites and early onset varicose veins. Other features include spontaneous pneumothorax, congenital hip dislocation, and/or talipes equinovarus. However, with increased availability of genetic testing, diagnoses of vEDS are also made in individuals who have few or no specific clinical features of vEDS on examination [25].

vEDS is usually caused by heterozygous pathogenic variants in *COL3A1* affecting the production of type III collagen, a major component of vessel walls and hollow organs. More than half of affected individuals have dominant negative substitutions for glycine in the repeated Gly-X-Y motif of the triple helical domain; the substituting amino acids are usually aspartate, arginine, valine or glutamate, whereas cysteine, serine, or alanine substitutions are underrepresented suggesting that these may potentially cause a milder phenotype. More than one third of individuals have splice-site and in-frame insertions–deletions in the triple-helical domain. Substitutions for glycine, splice site variants that result in in-frame deletion or insertion, and other in-frame variants are associated with a more severe clinical manifestation than less frequently observed null/LoF variants or non-glycine missense variants [17, 36]. Glutamate-to-lysine substitutions in triple helix domain Gly-Glu-Arg sequences have been reported in a number of families in which affected individuals showed prominent cEDS-type features in addition to vEDS complications [18]. Very occasionally, biallelic pathogenic variants in *COL3A1* may be identified, leading to a severe childhood-onset phenotype with structural brain anomalies. Several Arg>Cys variants in *COL1A1* (in particular p.Arg312Cys) have been reported to cause vascular fragility in combination with a (classical) vEDS phenotype [30]. Massive parallel sequencing of *COL3A1* (and *COL1A1*) – as always with deletion/duplication analysis – is expected to identify the causative pathogenic variant in >98 % of affected individuals.

## Rare EDS types associated with variants in collagen type I genes

Specific pathogenic variants in the type I collagen genes *COL1A2* or *COL1A1* may present with (classical) EDS features, sometimes overlapping with osteogenesis imperfecta-related bone fragility. Two distinct clinical entities have been defined in the current EDS classification.

### Cardiac-valvular EDS (cvEDS)

The term cvEDS is used for the very rare combination of an EDS phenotype with severe progressive cardiac-valvular disease, caused by biallelic null/LoF variants in *COL1A2* [40]. In the very few (<10) reported patients with cvEDS, severe mitral and/or aortic valve regurgitation necessitated valve replacement. However, complete absence of the proα2(I) chain encoded by *COL1A2* may cause variable clinical manifestations, including an osteogenesis imperfecta-EDS overlap syndrome associated with the production of a stable mutant proα2(I) chain that is degraded but may trigger an unfolded protein response (UPR) [7]. Genetic investigations in individuals with an EDS phenotype and severe cardiac valvular disease should entail thorough evaluation of the *COL1A2* gene, including large deletion analysis.

### Arthrochalasia EDS (aEDS)

This condition – originally described as “arthrochalasis multiplex congenita” [23] – has been reported as an EDS variant in approximately 50 individuals [7, 19]. aEDS usually presents at birth with extreme hypermobility and congenital bilateral hip dislocation, and is associated with recurrent subluxations and dislocations of both small and large joints. There may be various skeletal manifestations such as foot or spinal deformities, muscular hypotonia, and delayed motor development. Dysmorphic facial features include frontal bossing, hypertelorism, epicanthal folds, midfacial hypoplasia, depressed nasal bridge, and micrognathia. Most individuals also display the typical EDS features of soft, doughy, hyperextensible or redundant skin, often with easy bruising and atrophic scarring. aEDS is caused by complete or partial loss of exon 6 of either *COL1A2* or *COL1A1* that precludes trimming by procollagen N-proteinase and leads to retention of the N-propeptide in the collagen type I triple helix. Genetic investigations in individuals with suspected aEDS should focus on pathogenic *COL1A2* or *COL1A1* variants affecting exon 6 splicing and should include exon (5 and) 6 deletion analysis.

### COL1-related osteogenesis imperfecta/Ehlers-Danlos syndrome overlap disorder

This molecularly defined condition has not (yet) been classified as a specific EDS type. Affected individuals mainly present with severe joint hyperlaxity, soft and hyperextensible skin, abnormal wound healing, easy bruising, and sometimes signs of arterial fragility. In addition, they show subtle signs of osteogenesis imperfecta including blue sclerae, relatively short stature and osteopenia or fractures [31,35]. The diagnosis is based on the identification of pathogenic variants in *COL1A1* or *COL1A2*, often glycine substitutions affecting residues within or near the procollagen N-proteinase cleavage sites. Some individuals carry pathogenic variants previously reported as causative for osteogenesis imperfecta [35], indicating variable expressivity. The full molecular basis of this condition is not well understood, and the classification is open for discussion.

**Figure 1:**
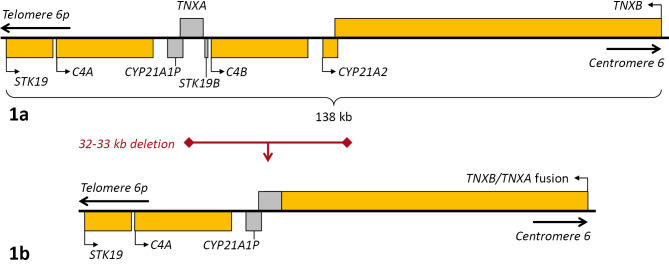
Genomic structure of the RCCX locus 1a: normal genomic structure; genes depicted in grey are pseudogenes. 1b: typical 32–33 kb deletion found in CAH-X syndrome

## Classical-like EDS (clEDS)

The term “classical-like” has been coined for phenotypes that resemble cEDS but are caused by pathogenic variants in non-collagen genes. Apart from the two genes discussed in more detail below, observations in a multi-generation family and a mouse model indicate that a heterozygous missense variant c.2686T>C (p.Cys896Arg) in *THBS2* may be another cause of clEDS [21]. Causality has not yet been confirmed in additional families, but in the meantime, it may be good to include *THBS2* as a possible clEDS gene in exome-based genetic analyses for EDS.

### Tenascin X-associated classical-like EDS (TNXB-clEDS, clEDS type 1)

The 2017 classification uses the term clEDS for the phenotype caused by complete loss of tenascin X (TN-X) function. This autosomal recessive condition – subsequently also called clEDS type 1 – resembles cEDS with generalized joint hypermobility, hyperextensible skin, and easy bruising, but is not associated with atrophic scarring. Other distinguishing features include mild muscle weakness, axonal polyneuropathy, leg oedema, and various anomalies of hands and feet including sometimes debilitating foot deformities. Organ prolapse and fragility, particularly of the gastrointestinal tract, is more frequent than in cEDS; gastrointestinal complications also include diverticulitis, gastrointestinal bleeding, intestinal obstruction, and gallstones. More than half of affected individuals report excessive fatigue [14, 20,46].

TN-X-deficient clEDS is caused by biallelic null/LoF variants of *TNXB* and has been reported in more than 50 individuals [46], but the diagnosis is technically challenging. Tenascin X is a large extracellular matrix glycoprotein that regulates deposition, stability and mechanical properties of collagen fibres [33]. The *TNXB* gene is a component of the complex RCCX locus in the major histocompatibility complex (MHC) class III region on chromosome 6q21.3. It comprises genes for serine/threonine kinase type 19 (*STK19,* previously denoted *RP*, and its pseudogene *STK19B*), complement 4 (two expressed genes *C4A* and *C4B*), steroid 21-hydroxylase (*CYP21A2* and its pseudogene* CYP21A1P*), and tenascin-X (*TNXB* and its pseudogene *TNXA*) [9] (Figure 1a). The homologous sequences of the locus are positioned in tandem in the same direction, leading to a propensity for non-allelic homologous recombination (NAHR). *TNXB* is a 68 kb gene with 44 exons, whereas the 4.5 kb non-transcribed *TNXA* pseudogene represents a 5’-truncated paralog and contains homologs to exons 32 to 44 only. NAHR-mediated deletion between *TNXB* and *TNXA* leads to loss of *CYP21A2*, the causal gene of autosomal recessive 21-hydroxylase deficiency (classical congenital adrenal hyperplasia, CAH), as well as *C4B,* absence of which may contribute to reduced complement 4 production. Furthermore, it results in distal *TNXB-TNXA* fusion that may have variable functional consequences (including possible abnormal protein-related effects in some heterozygotes) based on the exact recombination position and the extent of the inclusion of *TNXA*-specific variants [32].

Biallelic loss of functional *CYP21A2* in CAH is frequently caused by a large NAHR-mediated deletion on at least one allele. If the deletion results in loss of functional *TNXB* because of *TNXB-TNXA* fusion, the resulting phenotype combines CAH with Ehlers-Danlos syndrome, denoted CAH-X syndrome. Severity of the connective tissue manifestation may be linked to heterozygous or biallelic presence of the deletion, and potentially to specific variant TNX protein effects. Heterozygous loss of *TNXB* was claimed as a cause of hypermobile Ehlers-Danlos-Syndrome [48] but this association has not been confirmed. Population data indicate that TNXB is haplo*sufficient* (pLI = 0, LOEUF = 0,674, data from https://gnomad.broadinstitute.org). Due to the high sequence homologies, standard short-read sequencing methods do not allow determination of the exact genomic structure of the RCCX locus. A method combining long-range PCR with short-read sequencing for accurate detection of variants and *TNXA*-derived sequences in *TNXB* has been described [46]; long-read sequencing is also promising to resolve these issues. In consequence, the exact functional effects of genetic-genomic variants in the *TNXB* gene region and its relevance for hypermobility and different types of Ehlers-Danlos syndromes beyond clEDS remain to be clarified.

### AEBP1-associated classical-like EDS (AEBP1-clEDS, clEDS type 2)

Pathogenic variants in *AEBP1* as cause of clEDS were fully described only after the 2017 classification [1, 5], and the condition has also been called clEDS type 2. Affected individuals have the typical EDS manifestations of hypermobility, skin hyperextensibility, atrophic scarring and easy bruising, occasionally with other cardiovascular manifestations and/or hernias; a majority also has osteopenia sometimes with fractures, various skeletal abnormalities, and (mild) myopathy [3, 46]. *AEBP1* encodes two protein isoforms with various ECM and collagen-related functions. *AEBP1*-clEDS is an autosomal recessive condition caused mostly by biallelic null/LoF variants, and has been reported in >10 individuals. It is easily identifiable by standard (massively parallel) sequencing.

## EDS types associated with deficient collagen transcription regulation and processing

Deficiencies of proteins involved in transcription regulation or posttranslational processing of collagens or other ECM proteins are inherited as autosomal recessive traits. Beyond the trias of hypermobility, tissue fragility and skin hyperextensibility, they are often associated with – sometimes severe – skeletal manifestations, craniofacial dysmorphism and often specific sensory organ manifestations (eyes, hearing).

### Dermatosparaxis EDS (dEDS)

The clinical hallmark of dEDS is extreme fragility of generally redundant, lax and/or hyperextensible skin. Additional features include unusual craniofacial features (prominent eyes with puffy eyelids and excessive periorbital skin, postnatally large fontanels, a hypoplastic chin and bluish or greyish discoloration of the sclerae), easy bruising, postnatal growth retardation, and recurrent fractures. Joint hypermobility becomes more prominent with age. Prematurity and perinatal complications are common [7]. dEDS is an autosomal recessive condition repeatedly observed in animals (cattle, sheep, dogs, etc.) but only reported in <20 human individuals. It is caused by biallelic null/LoF variants in *ADAMTS2*, which encodes a disintegrin and metalloproteinase that cleaves the propeptides of type I and II collagens prior to fibril assembly. dEDS is easily identifiable by standard sequencing; there is one relatively frequent recurrent pathogenic variant c.673C>T (p.Gln225Ter) that has an allele frequency of 0.3 % in persons of Ashkenzi Jewish descent (gnomAD v4.1.0) [10].

### Kyphoscoliotic EDS (kEDS)

Children with kEDS often present at birth with marked muscular hypotonia and kyphoscoliosis without other neuromuscular abnormalities. Hypotonia tends to improve with age, whereas kyphoscoliosis is usually progressive and severe. There is generalized joint hypermobility, often associated with dislocations or subluxations of large joints (hips, shoulder, knees, wrist). The skin is hyperextensible, often with fragility and bruising. Additional skeletal abnormalities include deformities of hands and/or feet, as well as unusual craniofacial features. There are variable ocular manifestations such as bluish sclerae, microcornea or myopia; loss of an eye following trauma is a known risk in these individuals. Rupture of medium-sized arteries has been reported in several cases [7]. kEDS has been diagnosed in >100 individuals and is due to the autosomal recessive deficiency of either lysyl hydroxylase 1 encoded by *PLOD1,* which catalyzes the formation of hydroxylysine in collagens, or the peptidyl-prolyl cis-trans isomerase encoded by *FKBP14,* which assists processing of type III collagen in the endoplasmic reticulum and also interacts with types VI and X collagens. Individuals with *FKBP14*-kEDS often have impaired hearing, which is not usually observed in *PLOD1*-kEDS. Genetic testing for kEDS beyond standard sequencing should target a common 8.9 kb duplication of *PLOD1* exons 10–16 (c.1067_1846dup) that has a relative allele frequency of 30 %, i.e., represents 30 % of reported pathogenic alleles [7]. There is one relatively common *FKBP14* frameshift variant c.362dup (allele frequency 0,086 % in Europeans, gnomAD v.4.1.0) with a relative allele frequency of up to 70 % in European patients.

### Brittle cornea syndrome (BCS)

Marked corneal thinning with a high risk of corneal perforation in childhood – spontaneously or after minor trauma – is the leading manifestation of BCS. Typical ocular features prior to rupture include blue sclerae, keratoconus/keratoglobus, and high myopia. Most affected individuals have hypermobility predominantly of small joints; other skeletal anomalies such as kyphoscoliosis, foot deformities, developmental dysplasia of the hip or mild craniofacial abnormalities are common [7]. Skin abnormalities are milder than in other EDS types and may be absent. Hearing impairment is frequent. BCS has been reported in >60 individuals and is caused by biallelic (autosomal recessive) pathogenic (mostly null/LoF) variants in either *ZNF469* or *PRDM5*, which code for proteins involved in transcription regulation of collagens and/or other ECM proteins. BCS is easily identifiable by standard sequencing.

## EDS types caused by deficient glycosaminoglycan biosynthesis or ECM zinc maintenance

Glycosaminoglycans (GAGs) are long chains of sulfated or acetylated (amino) sugars attached to a protein skeleton via a tetrasaccharide linker region. They are major components of the viscous ECM, and through binding of a variety of ligands they contribute to the regulation of growth factor signalling and other cellular functions. Based on the carbohydrate sequence, heparan sulphate (HS), chondroitin sulphate (CS), and dermatan sulphate (DS, synthesized by modification of CS precursors) are distinguished. Deficiencies of enzymes required for biosynthesis and modification of GAG chains cause severe syndromic diseases characterized by skeletal dysplasia, joint hypermobility and contractures, variable skin and connective tissue manifestations, and sometimes intellectual disability. A similar phenotype is caused by the deficiency of a transporter protein for zinc, which is required for processing of collagens and other ECM proteins. All these conditions are inherited as autosomal recessive traits and are recognized by standard sequencing. Deficiencies of lysosomal enzymes required for GAG breakdown constitute the mucopolysaccharidoses.

### Spondylodysplastic EDS (spEDS)

Major clinical features of spEDS are progressive growth delay and short stature with bowing of limbs, other skeletal anomalies including osteopenia and recurrent fractures, and muscular hypotonia ranging from congenital severe to mild [7]. Affected children often show delayed cognitive and motor development, a characteristic facial gestalt, and joint hypermobility or contractures. The skin is often hyperextensible or loose, soft and translucent, but atrophic scars are rare, and there is usually no generalized tissue fragility. spEDS has been reported in >100 individuals and is inherited in an autosomal recessive fashion. It is mostly caused by biallelic null/LoF variants in enzymes required for tetrasaccharide linker assembly in GAG biosynthesis; known causative genes are *B4GALT7,* *B3GALT6,* and *B3GAT3*, encoding galactosyltransferase I, galactosyltransferase II, and glucuronyltransferase, respectively. The clinical manifestation shows minor differences between genes, and marked overlap with other defined phenotypes. The disorders of GAG linker assembly are often summarized as linkeropathies [37]. There is a prevalent *B4GALT7* founder variant c.808C>T (p.Arg270Cys) in the La Réunion island population, cause of the Larsen of La Réunion Island syndrome. Another form of spEDS, clinically somewhat different and originally denoted spondylocheirodysplastic EDS, is caused by biallelic (mostly null/LoF) variants in *SLC39A13.* This gene codes for the zinc transporter (importer) ZIP13, which is predominantly expressed in bone, teeth, and connective tissues [22].

### Musculocontractural EDS (mcEDS)

Congenital multiple contractures, characteristic craniofacial features, and subsequently EDS typical skin manifestations (hyperextensibility, fragility, atrophic scars, easy bruising) are the hallmarks of mcEDS. Joint dislocations, other skeletal changes (including spinal and thorax deformities), and various organ manifestations (myopia, hearing loss, heart defects, constipation, etc.) have been described [34]. This autosomal recessive condition has been independently recorded under different names such as “adducted thumb-clubfoot syndrome” or “Kosho type EDS”, and has been reported in >70 individuals. mcEDS is caused by the deficiency of enzymes required for the formation of dermatan sulphate, due to biallelic null/LoF variants in *CHST14* encoding dermatan 4-O-sulfotransferase 1, or less frequently in *DSE* encoding dermatan sulphate epimerase.

## EDS and myopathy

Muscular hypotonia is a prominent feature of several EDS types, and a number of primary muscle disorders are associated with joint hypermobility, highlighting the functional link between connective tissue integrity and muscle function [16, 43]. Ullrich congenital myopathy and the less severe Bethlem myopathy caused by pathogenic variants in the type VI collagen genes *COL6A1, COL6A2,* or* COL6A1,* are possibly the most prominent among the “myopathy and connective tissue overlap syndromes”; another example is *RYR1*-associated central core disease that frequently features generalized hypermobility and congenital hip dislocation. Genetic analysis in neonates with severe muscular hypotonia and evidence of joint hypermobility should target the relevant primary muscle disorders as well as the relevant connective tissue disorders.

### Myopathic EDS (mEDS)

The characteristic presentation of mEDS is neonatal muscular hypotonia associated with generalized joint hypermobility and (sometimes progressive) contractures [7, 13]. Severe cases may have fetal akinesia and require postnatal tube feeding; there are often various other skeletal anomalies. Motor development is usually delayed, muscle weakness tends to improve during childhood but may deteriorate again in adulthood. The skin tends to be soft and is sometimes hyperextensible; abnormal bruising or scarring is infrequent. mEDS has been reported in >20 individuals and is due to pathogenic variants in *COL12A1* that codes for the nonfibrillar homotrimeric type XII collagen. The severity of mEDS and the inheritance pattern in families depends on the functional effects of the individual variant(s): stable structural missense variants with a dominant negative effect are associated with autosomal dominant inheritance, whereas recessive inheritance has been reported for homozygous null/LoF variants. A large in-frame multi-exon deletion with severe heterozygous manifestation – suggesting a dominant negative effect – has been reported [12].

## Periodontal EDS (pEDS)

pEDS is characterized by severe and intractable periodontitis starting in childhood or adolescence, associated with lack of attached gingiva recognizable before onset of periodontitis. Additional features include easy bruising and pretibial plaques, mild mostly distal joint hypermobility, skin hyperextensibility, mild tissue fragility, and progressive mostly asymptomatic leukoencephalopathy-like alterations on brain imaging. The condition has been reported in approx. 200 individuals [4, 26]. pEDS is the only defined EDS type not caused by deficiency or alteration of collagens, collagen-processing/-regulating proteins, or other proteins with primary ECM functions. It is due to gain-of-function variants in the complement 1 protein genes *C1S* or *C1R* that trigger activation of the C1s serine protease. Activated C1s is able to degrade type I collagen (and other collagens), a probably desired function in the context of normal complement activation but the likely cause of the connective tissue pathology in pEDS [2]. The condition is easily identifiable by standard sequencing; null/LoF variants or large multi-exon deletions do not cause pEDS but in the biallelic state are possible causes of systemic lupus erythematosus [24].

## Genetic testing in individuals with suspected Ehlers-Danlos syndrome

The majority of individuals with “suspected EDS” have hypermobile EDS or hypermobility spectrum disorder, which at present cannot be confirmed or excluded by genetic tests. Generalized joint hypermobility has been recorded as clinical feature of various monogenic diseases beyond the defined EDS types. The OMIM disease database lists “hypermobility” in association with 189 different genes (www.omim.org, accessed July 1^st^ 2024). The Genomics England PanelApp (https://panelapp.genomicsengland.co.uk/panels/53/, accessed July 1^st^ 2024) lists 47 “green” genes (high level of evidence for gene-disease association) in the “Ehlers Danlos syndrome with a likely monogenic cause” category. This list contains a wide range of other connective tissue-associated disorders beyond the established EDS types, including various types of cutis laxa, Marfan and Loeys-Dietz syndromes, type VI collagen myopathies, and other diseases. Most of these conditions have specific clinical features that allow the reliable differentiation from the defined EDS types, and it is debatable whether the respective genes should be included in targeted “EDS panels”, particularly for adults. If one likes to cover these diagnoses because of the possibility of another monogenic connective tissue disorder in an individual with “suspected EDS”, it may be prudent to limit the extended analysis to (likely) pathogenic variants and not report potentially misleading VUS in non-EDS genes. This strategy needs to be well communicated to the referring colleagues.

**Figure 2:**
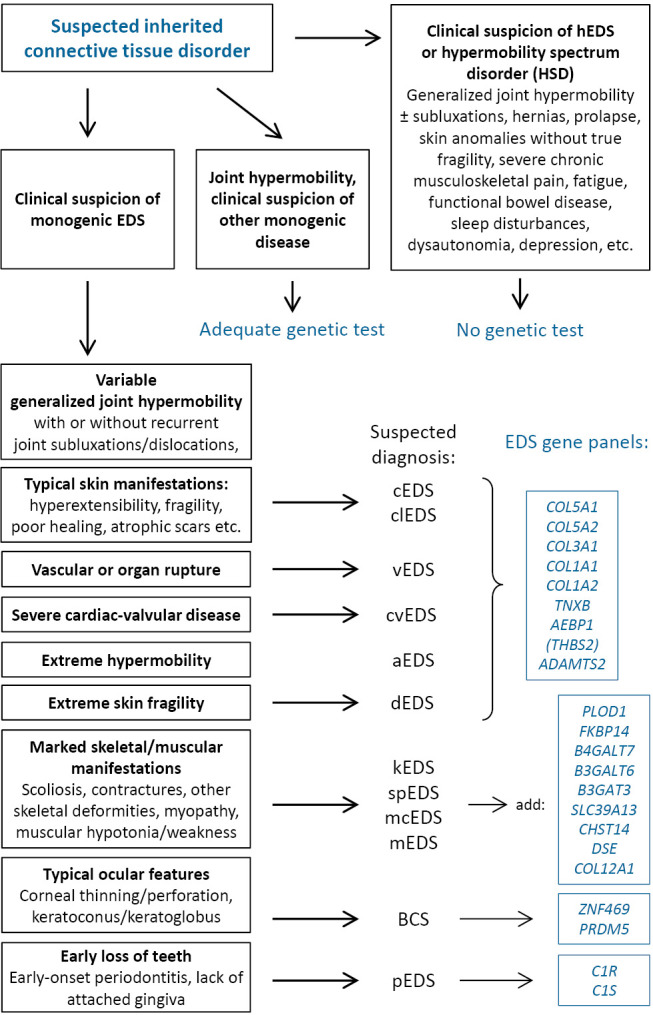
Genetic differential diagnosis of generalized joint hypermobility and suspected Ehlers-Danlos-Syndrome

To streamline genetic testing and to reduce VUS detection in unlikely relevant genes, thorough clinical evaluation is necessary prior to ordering exome- or genome-based analyses. Sequencing algorithms differ among countries based on factors including the availability of medical genetic expertise, laboratory flexibility, and financial resources. Figure 2 provides a suggestion for narrow targeted (virtual) gene panels in suspected monogenic EDS.

Diagnostic genetic testing is indicated in individuals with generalized joint hypermobility (with or without recurrent joint subluxations/dislocations) in conjunction with marked, typical skin manifestations (hyperextensibility, fragility, poor wound healing, atrophic scars, hemosiderosis etc.) and possibly arterial vascular events/organ rupture. In the absence of other pathognomonic skeletal, muscular, ocular, or oral manifestations, the genetic analyses may focus on the genes for type V, III and I collagen, *TNXB, AEBP1,* and *ADAMTS2* (*THBS2* unconfirmed).

In the case of marked scoliosis and/or other skeletal deformities, myopathy, and marked neonatal muscular hypotonia, the analyses should cover *PLOD1, FKBP14, B4GALT7,* *B3GALT6,* *B3GAT3, SLC39A13, CHST14,* *DSE* and* COL12A1. ZNF469* and *PRDM5* should be targeted if brittle cornea syndrome is a possibility, whereas analysis of *C1R* and *C1S* is only necessary in the presence of typical oral manifestations. Special considerations may apply for infants or children in whom the clinical features may not yet be fully developed.

Care must be taken to recognize EDS pathogenic variants that may be missed by standard exome-based variant calling, and it is prudent to provide the precise diagnosis or specific instructions to the diagnostic laboratory. Large multi-exon or whole gene deletions/duplications have been reported as causative for several EDS types; reliable quantitative analysis is particularly relevant for kEDS that is frequently caused by a duplication of *PLOD1* exons 10–16 (c.1067_1846dup). Careful assessment of different functional effects of quantitative (null/LoF vs. hypomorphic) or qualitative (e. g. dominant negative, gain of function) variant effects [47] is essential for the clinical interpretation of heterozygous variants in genes that are associated with dominant as well as recessive inheritance patterns. The complex genomic structure of the RCCX locus that contains *TNXB* and its exon 32–44 paralog *TNXA* hampers complete genetic analysis by short-read massive parallel sequencing. In the case of strong clinical suspicion of a particular EDS type and negative results of standard exome-based sequencing, the analyses may be extended to include the whole gene region (including introns, promotor and regulatory sequences) possibly in conjunction with transcript studies. International sharing of exome or genome variant data should assist in the identification of novel EDS-associated genes in the future.
